# Comparison of existing syncope rules and newly proposed anatolian syncope rule to predict short-term serious outcomes after syncope in the Turkish population

**DOI:** 10.1186/1865-1380-5-17

**Published:** 2012-04-20

**Authors:** Kamil Kayayurt, Haldun Akoglu, Onder Limon, Asım Oktay Ergene, Ozcan Yavasi, Serdar Bayata, Nergiz Vanden Berk, Erden Erol Unluer

**Affiliations:** 1 Department of Emergency Medicine, İzmir Atatürk Training and Research Hospital, Izmir, Turkey; 2 Department of Emergency Medicine, Dr. Lutfi Kirdar Kartal Training and Research Hospital, Istanbul, Turkey; 3 Department of Cardiology, İzmir Atatürk Training and Research Hospital, Izmir, Turkey

**Keywords:** clinical outcome, decision-making, emergency department, guidelines, prognosis, risk stratification, syncope

## Abstract

**Background:**

We wished to compare the San Francisco Syncope Rule (SFSR), Evaluation of Guidelines in Syncope Study (EGSYS) and the Osservatorio Epidemiologico sulla Sincope nel Lazio (OESIL) risk scores and to assess their efficacy in recognising patients with syncope at high risk for short-term adverse events (death, the need for major therapeutic procedures, and early readmission to the hospital). We also wanted to test those variables to designate a local risk score, the Anatolian Syncope Rule (ASR).

**Methods:**

This prospective, cohort study was conducted at the emergency department of a tertiary care centre. Between December 1 2009 and December 31 2010, we prospectively collected data on patients of ages 18 and over who presented to the emergency department with syncope.

**Results:**

We enrolled 231 patients to the study. A univariate analysis found 23 variables that predicted syncope with adverse events. Dyspnoea, orthostatic hypotension, precipitating cause of syncope, age over 58 years, congestive heart failure, and electrocardiogram abnormality (termed DO-PACE) were found to predict short-term serious outcomes by logistic regression analysis and these were used to compose the ASR. The sensitivity of ASR, OESIL, EGSYS and SFSR for mortality were 100% (0.66 to 1.00); 90% (0.54 to 0.99), 80% (0.44 to 0.97) and 100% (0.66 to 1.00), respectively. The specificity of ASR, OESIL, EGSYS and SFSR for mortality were 78% (0.72 to 0.83); 76% (0.70 to 0.82); 80% (0.74 to 0.85) and 70% (0.63 to 0.76). The sensitivity of ASR, OESIL, EGSYS and SFSR for any adverse event were 97% (0.85 to 1.00); 70% (0.52 to 0.82); 56% (0.40 to 0.72) and 87% (0.72 to 0.95). The specificity of ASR, OESIL, EGSYS and SFSR for any adverse event were 72% (0.64 to 0.78); 82% (0.76 to 0.87); 84% (0.78 to 0.89); 78% (0.71 to 0.83), respectively.

**Conclusion:**

The newly proposed ASR appears to be highly sensitive for identifying patients at risk for short-term serious outcomes, with scores at least as good as those provided by existing diagnostic rules, and it is easier to perform at the bedside within the Turkish population. If prospectively validated, it may offer a tool to aid physicians' decision-making.

## Introduction

Syncope is a symptom of cerebral hypoperfusion and is defined as a short, sudden, self-terminating episode of transient loss of consciousness with a failure to maintain postural tone and with a variety of aetiologies. More than two million people are evaluated for syncope each year in the US, with the cost running into the billions of dollars [1]. It accounts for approximately 3% to 5% of emergency department (ED) visits and, in selected patient populations, the lifetime prevalence of syncope could reach almost 50% [2,3].

Patients with syncope pose a difficult diagnostic dilemma. Most patients appear well and are asymptomatic on arrival, and there are often no witnesses to the event. Some patients will require emergency hospitalisation for a workup and for the treatment of life-threatening or potentially life-threatening causes [4,5]. Even after extensive inpatient evaluation, an underlying aetiology remains unknown in 30% to 50% of patients [6].

Clinical decision rules are tools designed to assist clinicians in making decisions at the bedside and to risk-stratify the patients. They are derived from original research and incorporate important predictors of outcome from the history, physical examination and basic diagnostic tests. The Osservatorio Epidemiologico sulla Sincope nel Lazio (OESIL) risk score, the San Francisco Syncope Rule (SFSR) and the Evaluation of Guidelines in Syncope Study (EGSYS) risk scores are largely used in emergency settings [7-9].

According to the OESIL study group, syncope score predictors of death after one year are an abnormal electrocardiogram (ECG), a history of cardiovascular disease (including heart failure), age of over 65 years old, and syncope without prodrome. Each risk factor counts as one point. In keeping with the OESIL study, we considered those patients who were characterised by a score of up to 1 to be low risk. Patients with a score of 2 or higher were assumed to be at intermediate or high risk (that is, they were admitted to the hospital) [7]. The SFSR was derived and validated to predict adverse outcomes at 7 and 30 days. Significant predictors of adverse events included a history of heart failure (CHF), an abnormal ECG (nonsinus rhythm on ECG or during ED monitoring, or new morphological changes on the ECG), a haematocrit of less than 30%, a complaint of shortness of breath, and a systolic blood pressure of less than 90 mmHg at triage [8]. According to the EGSYS research group, score factors associated with cardiac syncope include an abnormal ECG, a history of heart disease, palpitations prior to syncope, syncope while supine or during exertion, and the absence of an autonomic prodrome [9].

The aim of the present study was to compare the SFSR, the OESIL risk score and the EGSYS risk score to assess their efficacy in recognising patients with syncope at high risk for short-term (within seven days) adverse events (that is, death, the need for major therapeutic procedures, or early readmission to the hospital) and to test those variables to designate a local risk score, termed the Anatolian Syncope Rule (ASR). To the best of our knowledge, no comparative study for the Turkish population regarding the use of three clinical decision rules for syncope in a 7-day period and no proposed new decision rule have previously been published.

## Methods

This observational, single-centre, prospective cohort study was conducted in the ED of a research and training hospital. During a 13-month period, between December 1 2009 and December 31 2010, we prospectively collected data on patients who presented to the ED with syncope.

Patients were screened at triage on the basis of the following symptoms, findings and complaints for a potential syncopal event: syncope, loss of consciousness, presyncope, fainting, collapse, light-headedness, dizziness, falls, seizures, head injury and bone fractures. A research paramedic, who was blinded to the study protocol, ensured enrolment of all potential syncopal events. A patient evaluation form with the name of the patient written on it was brought to one of the five research physicians. The exclusion criteria used to determine the target population were as follows:

1. age below18;

2. unable to give either written or verbal informed consent;

3. confirmed nonsyncopal syndromes such as vertigo, coma, shock, witnessed seizure, sustained unconsciousness, head injury preceding loss of consciousness, stroke;

4. unable or unfeasible to follow-up (out of town residents, homeless);

5. presence of a clinical condition that required admission, such as acute myocardial infarction, pulmonary embolism, intracranial haemorrhage, sustained symptomatic bradycardia and tachycardia;

6. comorbidities with low survival rate;

7. pregnancy;

8. history of drug or alcohol abuse.

The final decision to enrol the patient was made by the research physicians and they completed the patient evaluation forms, containing approximately 70 variables that were derived from existing syncope rules, patient history, vital signs and physical examination. Where possible, attending physicians independently evaluated patients to measure agreement on subjective variables requiring interpretation. Only the attending physician assessments were used in the analysis and derivation of the model. All patients were treated in the usual manner by the attending physicians, who were blinded to the study protocol.

**Figure 1:**
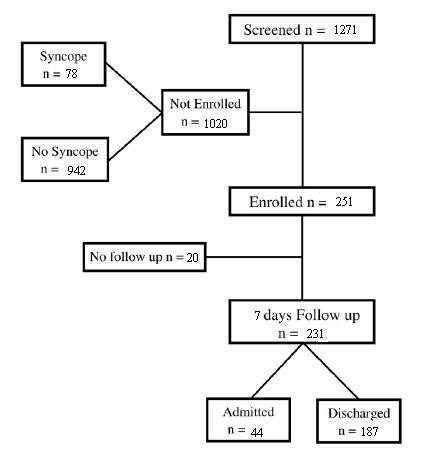
**Study population**.

As shown in Figure 1, a total of 1,271 consecutive patients were screened and 251 patients were enrolled. Twenty patients were lost to follow-up and 231 patients were eligible for the study.

The primary end-point of our study was the presence of any adverse event within seven days of discharge from the hospital, which included rehospitalisation, a major therapeutic intervention or death. No exceptions were made for these criteria. All discharged and admitted patients or their tutors were surveyed on seventh day of the index event by follow-up examination or interview and the presence of any adverse event was sought.

A major therapeutic intervention was defined as cardiopulmonary resuscitation, pacemaker or implantable cardioverter-defibrillator insertion, intensive care unit admittance or acute antiarrhythmic therapy. An ECG was defined as abnormal in the presence of any of the following in keeping with the criteria used in OESIL risk score: atrial fibrillation or tachycardia; a sinus pause of two seconds or more; sinus bradycardia with a heart rate ranging between 35 and 45 beats per minute; conduction disorders; ECG signs of previous myocardial infarction or ventricular hypertrophy; or multiple premature ventricular beats [10]. The ECG was analysed by the cardiologist on duty, who was unaware of the study and blinded to the three risk scores. We considered an ECG as abnormal independent of the onset time. Orthostatic hypotension was defined as a decrease of at least 20 mmHg in the systolic blood pressure or at least 10 mmHg in the diastolic blood pressure within two minutes of standing [6].

The study was approved by the Ethical Committee on Human Research of the Coordinating Centre, and participants provided written consent.

### Statistical analysis

SPSS version 15 (IBM, New York, USA) was used for descriptive data analysis. All continuous variables are presented as means with standard deviation (SD). Categorical variables are presented as percentages with 95% confidence intervals (CIs). A Pearson's chi-squared or Fisher's exact test was used for categorical variables, and a Student's *t*-test was used for continuous variables. To develop a score predictive of high-risk patients, binary variables were created from continuous variables. Univariate predictors of high-risk syncope were identified by chi-squared analysis. Variables with a high univariate correlation and with *P *< 0.10 were considered in stepwise logistic regression analyses with a cut-off *P-*value of < 0.05. Variables with similar meaning were combined to reduce the number of variables to be included in the logistic regression analysis (Table 1).

**Table 1:** Predictors of high-risk syncope on univariate analysis in the derivation cohort (n = 231)

Variable	Syncope without adverse events (n = 192) N (%)	Syncope with adverse events (n = 39) N (%)	*P*
Age > 58	68 (35.4)	32 (82.1)	< 0.001
Presence of tachycardia	23 (11.9)	9 (23.0)	> 0.05
Presence of tachypnea	5 (2.6)	9 (23.1)	< 0.001
Ortostatism	6 (3.1)	8 (20.5)	< 0.001
Co-morbidity	91 (47.4)	33 (84.6)	< 0.001
Polypharmacy	77 (40.1)	31 (78.5)	< 0.001
Abnormal physical examination finding	17 (8.9)	13 (33.3)	< 0.001
History of coronary artery disease	19 (9.9)	11 (28.2)	< 0.005
Presence of a prodrome	37 (19.3)	19 (48.7)	< 0.001
Abnormal ECG	31 (16.1)	28 (71.8)	< 0.001
History of congestive heart failure	9 (4.7)	13 (33.3)	< 0.001
Haematocrit < 30%	6 (3.1)	6 (15.4)	< 0.01
Presence of dyspnea	3 (1.6)	7 (17.9)	< 0.001
Presence of palpitations	15 (7.8)	8 (20.5)	< 0.05
Syncope on exertion	13 (6.8)	10 (25.6)	< 0.005
Syncope when supine	5 (2.6)	7 (17.9)	< 0.001
Any positive finding on auscultation	4 (2.1)	8 (20.5)	< 0.001
Abnormal ECG rhythm	8 (4.2)	8 (20.5)	< 0.005
Wide QRS	2 (1.0)	5 (12.8)	< 0.005
Abnormal ST (depression/elevation)	3 (1.6)	6 (15.4)	< 0.001
Abnormal cardiac axis on ECG	10 (5.2)	7 (17.9)	< 0.05
Presence of atrioventricular block	4 (2.1)	7 (17.9)	< 0.001
Known precipitating cause for syncope	96 (50)	34 (87.2)	< 0.001

ECG: electrocardiogram

Multivariable logistic regression analysis was then used to model the independent associations, demographic factors and whether any adverse event had happened, while controlling for sex and age as possible confounders. The goodness-of-fit for this regression model at each variable was verified by the Hosmer-Lemeshow test. The proportion of variance explained by the final model was determined using the Nagelkerke R statistics. The results of the multivariable logistic regression analysis were used to develop a risk score to predict the probability of any adverse event in seven days, named the ASR (Table 2). The relative associations of each variable as represented by their respective risk ratios were used to derive ASR (Table 2). Positive or negative values were assigned to each variable based on the relative magnitude of the regression coefficient. Then, the discriminative ability (ability of the score to classify patients and overall predictive performance) of the newly proposed ASR was calculated by measuring the area under the receiver operating characteristic (ROC) curve (AUC).

**Table 2:** Predictors of high-risk syncope from multivariate analysis with constant values of logistic regression and point scores for each predictor

Variable	*P *	Regression coefficient B (standard error)	Risk ratios	Score
(Constant)	< 0.001	-5.03 (0.72)		
**D**yspnoea	< 0.005	3.04 (0.97)	4.83	1
**O**rtostatism	< 0.05	1.60 (0.80)	4	1
**P**recipitating cause for syncope	< 0.005	1.78 (0.61)	5.28	1
**A**ge > 58	< 0.05	1.45 (0.58)	5.99	1
**C**ongestive heart failure history	< 0.05	1.47 (0.61)	4.75	1
**E**lectrocardiogram	< 0.001	1.72 (0.53)	7.42	2

Note: R^2 ^= 0.33 (Cox and Snell), 0.56 (Nagelkerke). Model Chi-square (1) = 93.08, *P *< 0.001.

We compared the prognostic accuracy of ASR and other risk scores by generating ROC curves and comparing the AUC using the method described by Hanley and McNeil for comparing ROC curves derived from the same cases (MedCalc Software version 10.4.0.0; MedCalc, Mariakerke, Belgium). When assessing potential decision thresholds in each respective risk score, our goal was to achieve near 100% sensitivity for need for intervention or death at the highest possible specificity. According to this criterion, we dichotomized ASR as high- and low-risk values from the decided thresholds for any adverse event and for mortality. Then, we calculated sensitivity, specificity, positive predictive value, negative predictive value, positive likelihood ratio and negative likelihood ratio values for any adverse events for each syncope criterion, including the criteria that we had defined (ASR). To determine the concordance of high- and low-risk criteria with the presence of any adverse event, kappa values for each criterion were calculated.

A two-tailed *P *< 0.05 was considered to be statistically significant for all analyses.

## Results

**Figure 2:**
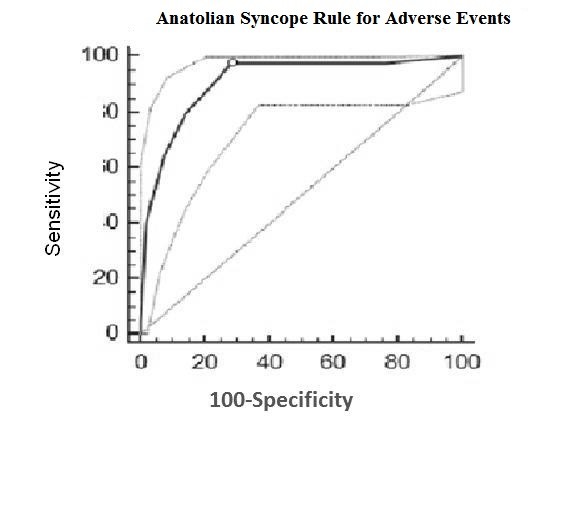
**Receiver operating characteristic analysis for predicting any adverse event**. ROC analysis of the newly proposed Anatolian Syncope Rule in the cohort for predicting any adverse event. Dotted lines define the 95% confidence intervals.

**Figure 3:**
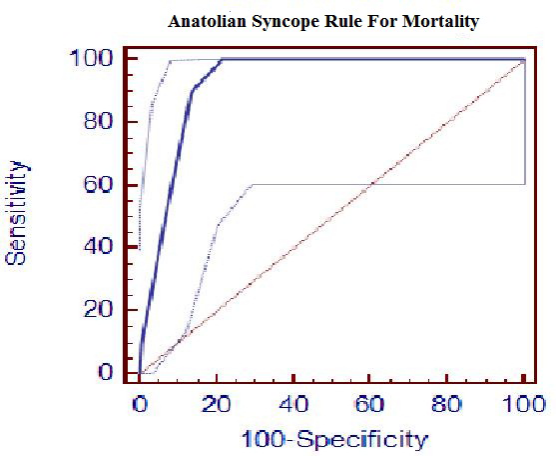
**Receiver operating characteristic analysis for predicting any mortality**. ROC analysis of the newly proposed Anatolian Syncope Rule in the cohort for predicting any mortality. Dotted lines define the 95% confidence intervals.

Six parameters (presence of dyspnoea, orthostatic hypotension, any precipitating cause of syncope, age over 58 years old, presence of congestive heart failure, and ECG abnormality) were found to have significant predictive value according to logistic regression analysis. They were represented by a simple mnemonic, DO-PACE, to compose the newly proposed ASR (Table 2). Precipitating factors were drugs, diabetes and neurologic disorders in patients with orthostatic syncope (40% of the known precipitating group). Fever, dehydratation, fasting and long standing were the precipitating factors in vasovagal syncope (45% of the known precipitating group). The other 15% were due to arrhythmia and cardiogenic causes. Figure 2 shows the ROC analysis of the newly proposed ASR scores for adverse events in syncope derived from the risk ratios. The AUC was 0.909 (95% CI: 0.864 to 0.942), indicating a good discriminant ability. A point score > 1 was considered the best discriminator for a diagnosis of high-risk syncope. The newly proposed ASR performed better, with a higher kappa scores and higher sensitivity (Table 3). The same calculations were performed to detect the discriminative power of each criterion when predicting mortality (Table 4). The AUC was 0.927 (95% CI: 0.885 to 0.957), indicating good discriminant ability. A point score > 2 was considered the best discriminator for a diagnosis of high-risk syncope that resulted in mortality. The newly proposed ASR performed with higher sensitivity but lower specificity when predicting mortality, assuming a cut-off score > 1. However, ROC analysis (Figure 3) showed that the cut-off value with the highest discriminative ability was > 2 for mortality (Table 4). With this new cut-off for mortality, the newly proposed ASR outperformed the other diagnostic criteria.

**Table 3:** Sensitivity, specificity, positive predictive value, negative predictive value, positive likelihood ratio, negative likelihood ratio and Kappa values for each syncope criteria for any adverse event

	OESIL (95% CI)	SFSR (95% CI)	EGSYS (95% CI)	ASR (95% CI)
**Sensitivity**	0.70 (0.52 to 0.82)	0.87 (0.72 to 0.95)	0.56 (0.40 to 0.72)	0.97 (0.85 to 1.00)
**Specificity**	0.82 (0.76 to 0.87)	0.78 (0.71 to 0.83)	0.84 (0.78 to 0.89)	0.72 (0.64 to 0.78)
**True positive** **(PPV)**	0.44 (0.32 to 0.57)	0.44 (0.33 to 0.56)	0.42 (0.29 to 0.57)	0.41 (0.31 to 0.52)
**False positive**	0.56 (0.42 to 0.68)	0.56 (0.44 to 0.67)	0.57 (0.43 to 0.71)	0.59 (0.48 to 0.68)
**True negative** **(NPV)**	0.93 (0.88 to 0.96)	0.97 (0.92 to 0.99)	0.91 (0.85 to 0.94)	0.99 (0.95 to 1.00)
**False negative**	0.07 (0.04 to 0.12)	0,03 (0.01 to 0.08)	0.09 (0.06 to 0.15)	0.007 (0.00 to 0.05)
**Positive LR**	3.91 (2.70-5.66)	3.89 (2.91-5.20)	3.61 (2.35-5.55)	3.46 (2.75-4.37)
**Negative LR**	0.37 (0.23-0.60)	0.17 (0.07-0.38)	0.52 (0.36-0.74)	0.03 (0.005-0.25)
**Kappa for adverse events**	0.44	0.52	0.37	0.53

ASR: Anatolian Syncope Rule; CI: confidence interval; EGSYS: Evaluation of Guidelines in Syncope Study; LR: likelihood ratio; NPV: negative predictive value; OESIL: Osservatorio Epidemiologico sulla Sincope nel Lazio; PPV: positive predictive value; SFSR: San Francisco Syncope Rule.

**Table 4:** Sensitivity, specificity, positive predictive value, negative predictive value, positive likelihood ratio, negative likelihood ratio and Kappa values for each syncope criteria for mortality

	OESIL (95% CI)	SFSR (95% CI)	EGSYS (95% CI)	ASR > 1 (95% CI)	ASR > 2 (95% CI)
**Sensitivity**	0.90 (0.54 to 0.99)	1 (0.66 to 1.00)	0.80 (0.44 to 0.97)	1 (0.66 to 1)	1 (0.66 to 1)
**Specificity**	0.76 (0.70 to 0.82)	0.70 (0.63 to 0.76)	0.80 (0.74 to 0.85)	0.63 (0.56 to 0.69)	0.78 (0.72 to 0.83)
**True positive** **(PPV)**	0.15 (0.07 to 0.27)	0.13 (0.07 to 0.23)	0.15 (0.07 to 0.29)	0.11 (0.06 to 0.20)	0.17 (0.09 to 0.30)
**False positive**	0.85 (0.74 to 0.93)	0.87 (0.77 to 0.93)	0.85 (0.71 to 0.93)	0.89 (0.80 to 0.94)	0.83 (0.70 to 0.91)
**True negative** **(NPV)**	0.99 (0.96 to 1.00)	1 (0.97 to 1.00)	0.99 (0.96 to 1.00)	1 (0.97 to 1)	1 (0.97 to 1)
**False negative**	0.006 (0.00 to 0.04)	0 (0.00 to 0.03)	0.01 (0.00 to 0.04)	0 (0 to 0.03)	0 (0 to 0.03)
**Positive LR**	3.83 (2.79 to 5.24)	3.30 (2.70 to 4.03)	4.02 (2.67 to 6.04)	2.70 (2.27 to 3.20)	4.60 (3.58 to 5.91)
**Negative LR**	0.13 (0.02 to 0.84)	0 (0 to 0)	0.25 (0.07 to 0.86)	0 (0 to 0)	0 (0 to 0)
**Kappa for** **mortality**	0.31	0.30	0.29	0.26	0.37

ASR: Anatolian Syncope Rule; CI: confidence interval; EGSYS: Evaluation of Guidelines in Syncope Study; LR: likelihood ratio; NPV: negative predictive value; OESIL: Osservatorio Epidemiologico sulla Sincope nel Lazio; PPV: positive predictive value; SFSR: San Francisco Syncope Rule.

## Discussion

Despite the limitations of our study, to the best of our knowledge this is the first reported cohort study of syncope patients to compare all three clinical rules and the only attempt to propose a clinical decision rule to identify high-risk patients from the Turkish population. The predictor variables used in the evaluation of patients with syncope developed a highly sensitive clinical decision rule that we believe will augment physician judgement and allow physicians to decide rationally which patients with syncope need admission according to their risk for short-term serious outcomes. The rule is not complex and easily remembered by a simple mnemonic: DO-PACE (Dyspnoea, Orthostatic hypotension, Precipitating cause of syncope, Age over 58 years old, Congestive heart failure, and ECG abnormality).

The demographic variables of age, sex, and race are potential risk factors for cardiovascular disease. Epidemiologic and cohort studies have confirmed the importance of age; however, of course, age alone is a marker for increased mortality. Although increasing age is accompanied by an increased risk of poor outcomes, there is no single cut-off for age but rather a continuum of gradually increasing risk [11-13]. The OESIL study found that an age of over 65 years is one of the predictors. However, in our decision rule, an age of over 58 years is one of the predictors of high-risk syncope. We used ROC analysis to find the best age cut-off to discriminate between having an adverse event or not. Age 58 years was found to have the highest accuracy with the highest sensitivity and specificity and therefore was chosen to be the cut-off of the newly proposed ASR (sensitivity 82.05% (95% CI: 66.5 to 92.5); specificity 64.58% (95% CI: 57.4 to 71.3)). If we had used the age of 65 as the cut-off in our study, as in the OESIL study, our sensitivity would have dropped, and specificity would have increased (sensitivity 56.41% (95% CI: 39.6 to 72.2); specificity 73.96% (95% CI: 67.1 to 80.0)). Additionally, we would have misgrouped 11 patients (22.4% of the patients with any adverse event) as low risk. To include any patient with any adverse event, the cut-off age to be chosen would have been 40 (sensitivity 100.00% (95% CI: 91.0 to 100.0); specificity 38.02% (95% CI: 31.1 to 45.3)). However, we would have grouped seven more patients correctly as high risk (14%) at the expense of grouping 53 patients (27.6% of the patients without any adverse events) as high risk when they actually were not. Nevertheless, there were no high-risk patients between the ages of 40 and 58 years old who were misgrouped as low risk just because of their ages. All of the patients who ended up with adverse events had another positive risk factor, other than their age, that grouped them into the high-risk category according to the newly proposed ASR. In conclusion, the age of 58 years old was the best-fit cut-off for our purposes, and when used in the newly proposed ASR, the diagnostic abilities are the same as when either 40 or 58 years old is chosen as a cut-off.

Orthostatic hypotension may identify some patients with syncope related to volume depletion, autonomic insufficiency or medications. Orthostatic hypotension is common in patients with syncope of unknown aetiology, and this finding is also present in up to 40% of asymptomatic patients older than 70 years old and 23% of those patients younger than 60 years old [14]. A diagnosis of orthostatic hypotension should be a diagnosis of exclusion in otherwise low-risk patients, with the realisation that many high-risk patients will have orthostasis [15]. In our cohort, orthostatic hypotension was one of the predictors of adverse events in both the univariate and multivariate analysis that had not been described before in any other decision rule.

Sarasin *et al. *showed that, in patients with syncope, ST-segment and T-wave abnormalities were one of the significant predictors of arrhythmias [16]. In another study, the presence of an abnormal ECG, excluding a nonspecific ST-segment and T-wave changes, was a multivariate predictor for arrhythmia or death within one year after the syncopal episode [17]. Arrhythmia-related syncope is diagnosed by ECG when there is sinus bradycardia < 40 beats/min or repetitive sinoatrial blocks or sinus pauses > 3 sec; a Mobitz II 2nd- or 3rd-degree atrioventricular block; an alternating left and right bundle branch block; a rapid paroxysmal supraventricular tachycardia or ventricular tachycardia; or pacemaker malfunction with cardiac pauses [18]. In our study, tachycardia, the presence of palpitations, an abnormal ECG or abnormal rhythm, a wide QRS complex, an abnormal ST deviation, an abnormal cardiac axis and the presence of an atrioventricular block were the predictors of high-risk syncope in univariate analysis. We combined all ECG-related predictors into one, and 'abnormal ECG' defines all of them in the logistic regression analysis.

In a prospective study in Europe of 676 patients with syncope, predictors of short-term unfavourable outcome at 10 days included a mean age of 59 years old, abnormal ECG findings, concomitant trauma, an absence of prodromal symptoms and male sex. The occurrence of long-term unfavourable outcome was correlated with an age older than 65 years, a history of neoplasms, cerebrovascular disease, structural heart diseases and ventricular arrhythmias. These investigators concluded that risk factors for short- and long-term adverse outcomes after syncope were different and that long-term mortality was related to the comorbidities. Significant independent prognostic factors for time to mortality were diabetes, coronary artery bypass graft surgery, a history of malignancy, narcotics use, smoking, volume depletion and atrial fibrillation [19]. In our study, the presence of prodromal symptoms, coronary artery disease and congestive heart disease were the predictors of high-risk syncope in univariate analysis.

A recent study by Quinn *et al. *defined multiple univariate predictors of serious outcomes that included an abnormal ECG, a haematocrit of less than 30%, a complaint of shortness of breath, hypotension and a prior history of congestive heart failure [7,12]. In our study, tachypnoea, dyspnoea and history of congestive heart failure were the predictors of high risk and were correlated with the literature.

An absent or brief prodrome (less than 5 seconds) may be present with dysrhythmias, whereas neurally mediated syncope (synonyms include neurocardiogenic syncope and vasovagal syncope) may be characterised by longer prodromes and associated with nausea or vomiting [11].

When developing any prediction rule, one can always achieve 100% sensitivity but often at the expense of specificity and by overfitting of the statistical model. For example, in our model, we could have achieved 100% sensitivity by adding an age older than 40 years old to the rule, which would have identified the seven patients not predicted by the rule. We thought that the trade-off was suboptimal because the absolute admission rate for patients deemed to be high risk would be the same as, if not slightly higher than, at baseline. We were also aware that rare, serious outcomes not in our derivation set would make validating any rule with 100% sensitivity and certainty almost impossible. We thus accepted a rule that maximised sensitivity and specificity at the expense of not achieving 100% sensitivity. We purposely did not develop the rule with the physician's decision-to-admit as the primary outcome. Thus, this rule is not a decision rule to predict admission. The reasons to admit often take other factors (for example, social factors) into consideration that, although important, we believed were not specific to whether patients with syncope are at an acute risk for serious outcomes that require acute hospitalisation.

### Study limitations

Our study has some limitations. First of all, power calculations were not made prior to commencement of the study because there were no published articles to guide our three-way comparison. This was a single-centre study, thus, our patient group may not represent the whole Turkish population. Attending physicians reviewed all cases independently and the decisions of attending physicians superseded in case of dispute. However, inter-rater reliability of all subjective findings was reasonable, fair or worse with kappa values of 0.5 or less. So we decided to accept attending senior physicians' decisions for the analysis and derivation of the model.

## Conclusions

This prospective cohort study has shown that many variables included here, but not described before, are associated with serious outcomes in patients with syncope. SFSR performed better in the prediction process of any short-term adverse events. SFSR also had the best score to rule-out short-term mortality. However, none of these scores either rule-in or rule-out any short-term adverse events or mortality well enough to be suggested. The newly proposed ASR appears to be highly sensitive for identifying patients at risk for short-term serious outcomes, at least as good as other scores, and it is easier to perform at the bedside for the Turkish population. If prospectively validated, it may offer a tool to aid physicians' decision-making. Our proposed decision rule now needs to be validated prospectively in a large cohort of patients before physicians can consider it in clinical decision-making.

## Competing interests

The authors declare that they have no competing interests.

## Authors' contributions

KK made substantial contributions to the conception and design, acquisition of data and interpretation of data. HA made substantial contributions to the conception, design, and analysis and interpretation of data, and was involved in drafting the manuscript. OL made substantial contributions to the design, the analysis and interpretation of data, and was involved in drafting the manuscript. AOE made substantial contributions to the conception and design, and the analysis and interpretation of data. OY contributed to the analysis and interpretation of data, and was involved in drafting the manuscript. SB made substantial contributions to the conception and design, and analysis and interpretation of data. NVB made substantial contribution to the conception and interpretation of the data and was involved in drafting the manuscript. EEU made substantial contributions to the conception and design, and analysis and interpretation of data. All authors read and approved the final version of this manuscript.
